# The desire to die in palliative care: a sequential mixed methods study to develop a semi-structured clinical approach

**DOI:** 10.1186/s12904-020-00548-7

**Published:** 2020-04-16

**Authors:** Kerstin Kremeike, Gerrit Frerich, Vanessa Romotzky, Kathleen Boström, Thomas Dojan, Maren Galushko, Kija Shah-Hosseini, Saskia Jünger, Gary Rodin, Holger Pfaff, Klaus Maria Perrar, Raymond Voltz

**Affiliations:** 1 Department of Palliative Medicine, Medical Faculty, University of Cologne, Kerpener Str. 62, 50937 Cologne, Germany; 2 Institute of Medical Statistics and Computational Biology, Medical Faculty, University of Cologne, Cologne, Germany; 3 Cologne Center for Ethics, Rights, Economics, and Social Sciences of Health (ceres), University of Cologne and University Hospital of Cologne, Cologne, Germany; 4 Department of Supportive Care, Princess Margaret Cancer Centre, University Health Network, Toronto, Canada; 5 Institute of Medical Science, University of Toronto, Toronto, Canada; 6 Department of Psychiatry, University of Toronto, Toronto, Canada; 7 Institute of Medical Sociology, Health Services Research, and Rehabilitation Science (IMVR), University of Cologne, Medical Faculty, Cologne, Germany; 8 Center for Integrated Oncology Aachen Bonn Cologne Düsseldorf (CIO ABCD), University of Cologne, Cologne, Germany; 9 Clinical Trials Centre Cologne (ZKS), University of Cologne, Cologne, Germany

**Keywords:** Desire to die, Wish towards hastened death, Suicidal ideation, relationship, communication, Palliative care, Professionals, Consensus, Patients, Germany

## Abstract

**Background:**

Although desire to die of varying intensity and permanence is frequent in patients receiving palliative care, uncertainty exists concerning appropriate therapeutic responses to it. To support health professionals in dealing with patients´ potential desire to die, a training program and a semi-structured clinical approach was developed. This study aimed for a revision of and consensus building on the clinical approach to support proactively addressing desire to die and routine exploration of death and dying distress.

**Methods:**

Within a sequential mixed methods design, we invited 16 palliative patients to participate in semi-structured interviews and 377 (inter-)national experts to attend a two-round Delphi process. Interviews were analyzed using qualitative content analysis and an agreement consensus for the Delphi was determined according to predefined criteria.

**Results:**

11 (69%) patients from different settings participated in face-to-face interviews. As key issues for conversations on desire to die they pointed out the relationship between professionals and patients, the setting and support from external experts, if required. A set of 149 (40%) experts (132/89% from Germany, 17/11% from 9 other countries) evaluated ten domains of the semi-structured clinical approach. There was immediate consensus on nine domains concerning conversation design, suggestions for (self-)reflection, and further recommended action. The one domain in which consensus was not achieved until the second round was “proactively addressing desire to die”.

**Conclusions:**

We have provided the first semi-structured clinical approach to identify and address desire to die and to respond therapeutically – based on evidence, patients’ views and consensus among professional experts.

**Trial registration:**

The study is registered in the German Clinical Trials Register (DRKS00012988; registration date: 27.9.2017) and in the Health Services Research Database (VfD_DEDIPOM_17_003889; registration date: 14.9.2017).

## Background

Desire to die is a complex phenomenon with individual reasons, forms and consequences [1, 2]. We use the term desire to die in a broad sense including an acceptance of death, a wish for hastened death without requiring any accelerating action [3], a request for assisted dying and suicidal ideation [4]. This understanding of desire to die differs from the international consensus definition of the wish for hastened death [5] and refers to the German palliative care guideline for patients with incurable cancer [6]. The broad definition is meant to foster a more open communication with patients as it supports health professionals’ acceptance of desire to die as a potential way to cope with a terminal illness.

Desire to die can coexist with a simultaneous will to live [7], with both prone to change over time [1, 8]. Desire to die is connected with physical and psychological distress [9] and can be the beginning of a suicidal process [10], but studies have shown that communication concerning therapeutic options may ease patient’s burden, and even prevent suicides [11]. Current recommendations suggest proactively addressing desire to die [6], referencing studies which found positive effects on patients [8, 12, 13]. These effects include opening communication about emotional conditions, even in absence of desire to die [13]. Additionally, desire to die may also be assessed by validated instruments such as the Schedules of Attitudes for Hastened Death (SAHD; primary for research purposes) [8] or the Desire for Death Rating Scale (DDRS; initially developed for clinical interviews) [14].

Some forms of desire to die are frequent in patients in their last months of life. In a 1995 study, 45% of 200 advanced cancer patients showed at least occasional desire to die and almost 10% reported a strong and persistent one [15]. A recent survey of 377 cancer patients found that 18% reported an occasional desire to die and 12% a serious one [16]. Although health professionals are frequently confronted with a patient’s desire to die [3], it is not routinely assessed in palliative care. A lack of preparation on how to deal with the complex and sensitive topic as well as the controversial German legal situation contribute to uncertainty in health professionals about how to approach desire to die in clinical practice [17]. Both euthanasia ("termination of life on request"; § 216 national criminal code) and physician-assisted suicide ("assistance of suicide with intent of repeated conduct; § 217 national criminal code) were prohibited in Germany at the time of the study. As a consequence, patients who desire physician-assisted suicide may have travelled to neighboring countries with less restrictive regulations [18]. Between 2008 and 2012, nearly half (44%) of all so called “suicide tourists” in Switzerland came from Germany [18]. Nevertheless, 74% of German doctors stated in 2017 that they had been asked by their patients to assist suicide [19]. In February 2020, the Federal Court of Justice declared the prohibition of assistance of suicide with the intent of repeated conduct to be inadmissible, thereby repealing §217 [20]. The court decided this law introduced in 2015 to violate the German Constitution; there is a right to die in a self-determined way including the freedom to take one’s own life and to take advantage of offers from third parties. It remains to be seen what consequences this ruling will have. What is certain is that it will continue to be the subject of controversial discussions.

A restrictive as well as unclear legal situation in combination with a lack of preparation and knowledge can lead health professionals to neglect or insufficiently discuss desire to die [17], even if raised by the patient. Training programs and recommendations for talking about difficult issues [21, 22] may also support health professionals in dealing with desire to die [23]. A training program regarding desire to die has been developed in a previous project, based on a literature review and results of focus groups with multi-professional German palliative care providers [24]. This training program has been piloted and evaluated through a 32-item-scale covering the dimensions of self-confidence, skills, knowledge and attitudes [25]. Descriptive changes indicate a major improvement in self-confidence and at least minor improvements on all dimensions after 3 months, with only one item concerning knowledge reaching statistical significance, though [24]. Within the development of the training program, a semi-structured clinical approach was drafted, building on published recommendations on dealing with desire to die [26, 27] and based on results from an interdisciplinary advisory board discussion. The draft was refined and structured within a framework analysis approach [24]. The present study aimed at further developing and refining the clinical approach for routinely assessing death and dying distress, reacting to and (proactively) addressing desire to die – taking into account international expertise from a wider range of professions, patient representatives and relatives as well as the voices of patients.

## Methods

**Fig. 1:**
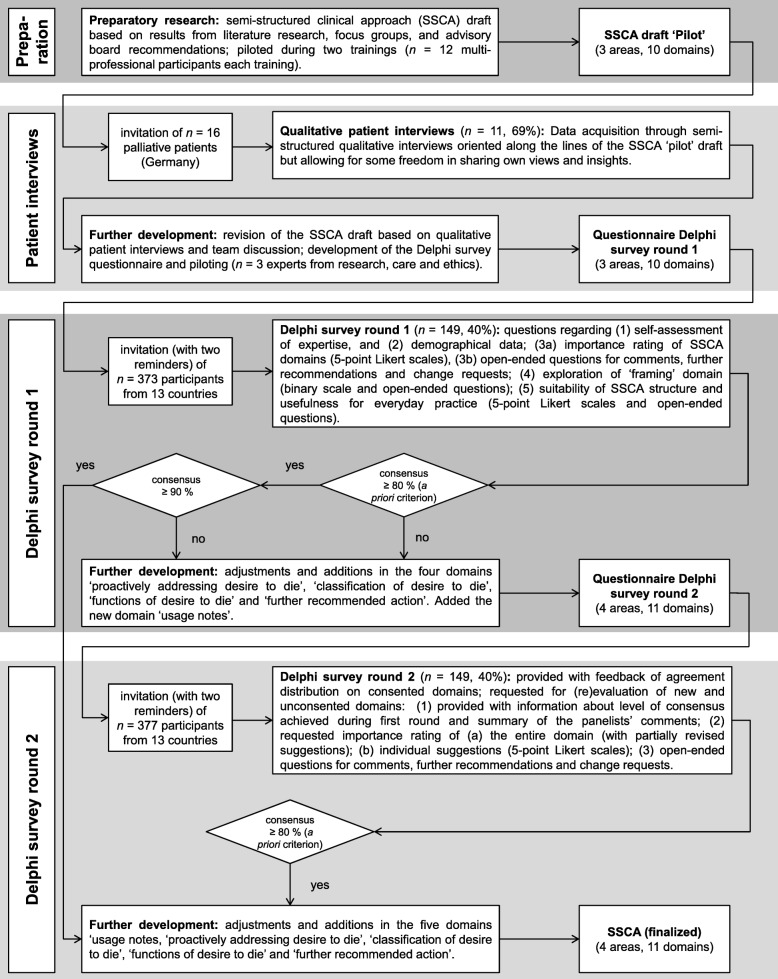
Flowchart of the semi-structured clinical approach development process

A sequential mixed methods design was used including qualitative patient interviews and a Delphi survey with experts (see Fig. 1). Both methods were chosen to maximize the approach’s clinical relevance. More details on the background on and justification of the methods selected are described in the study protocol [28]. We applied the consolidated criteria for reporting qualitative research (COREQ) [29] and the guidelines for conducting and reporting Delphi Studies (CREDES) in palliative care [30]. Research was conducted according to the Declaration of Helsinki. Ethical approval for this study was obtained from the Ethics Committee of the University of Cologne (#17–265).

### Patient interviews

Interviews were conducted to consider the perspective of patients receiving palliative care and thereby strengthen the appropriateness of the approach. A convenience sample of 16 adult patients was invited to participate in face-to-face interviews on the appropriate approach to desire to die in palliative care. Participants were recruited via palliative care providers known to the research team. A leaflet for patients and health professionals included survey aims, procedure and contact details of the research team. Health professionals contacted the researchers in case of patients’ willingness to participate. The research team approached these patients to obtain written informed consent for study participation. All data relating to the patients were anonymized to protect their identity and prevent retrospective matching of persons and information.

Interviewees chose the location of the interview, which were conducted by either one of three female (KK, VR, KB) or one male (GF) interviewers. Two researchers hold doctoral degrees (KK, GF), one was a doctoral student (VR), the other a graduate student (KB). At the time of data acquisition, all researchers worked for the Department of Palliative Medicine at the University Hospital of Cologne and were trained and experienced in conducting semi-structured interviews. This approach to data acquisition allowed the research agenda to be pre-defined to some extent and at the same time enabled respondents to freely present a range of views and new insights [31]. The interview guide was developed by a group of social scientists (KK, GF, MG), psychologists (TD, KB), a pedagogue (VR) and a physician (KMP). It assessed aspects relevant to addressing desire to die, desirable traits in interviewer as well as interviewees’ potential personal desire to die. The complete interview guideline is added as an additional file (see Additional file 1). Social demographics were assessed using a brief questionnaire.

### Delphi survey

In addition to patient interviews, an online Delphi survey was performed to achieve expert consensus on content and structure of the clinical approach. We invited participants from 13 countries aiming to compose a balanced sample of 50–70 researchers and clinical practitioners. Recruitment took place through personal contacts and an internet search. Potential participants were asked via email to take part in the survey and to propose further experts. As nurses were initially underrepresented in recruitment, we reached out to the nursing mailing list of the German Society for Palliative Medicine, which distributed our call to a selection of its members. This pushed the number of invited participants to 377.

To develop the Delphi questionnaire, we revised the existing version of the semi-structured clinical approach [24] based on our preceding patient interviews. The revised draft included three sections (B to D in Table 3) with a total of ten domains each containing a set of suggestions for health professionals to take into account when (proactively) discussing desire to die with patients receiving palliative care. The Delphi survey was designed accordingly and conducted in co-operation with an external partner (UZ Bonn, Society for Empirical Social Research and Evaluation). Each panelist received a link to the survey via email. During both Delphi rounds two reminders were sent out to motivate non-respondents to participate. During the first Delphi round, participants evaluated each domain of the approach by a) rating the importance of the entire section presented on a five-point Likert scale ranging from 0 (“not important at all”) to 5 (“very important”) and b) giving free text comments on the suggestions listed in the domain they just rated. In the second Delphi round, participants were asked to reevaluate the semi-structured approach in light of the results of the first round. Participants were also fed back information about the first rounds’ sample seize such as distribution of professions and the sample’s experience with desire to die and suicidality.

### Data analysis

Patient interviews were transcribed verbatim and analyzed by three researchers (KB, KK, GF) applying qualitative content analysis [32]. Inductive and deductive categories were derived and applied using the qualitative data analysis software MAXQDA 12. Inter-coder reliability was ensured by constant comparison between coders and according adjustments throughout the entire process. Transcripts were not returned to participants and they were not asked to provide feedback on the findings.

Delphi panelists’ data was eligible for analysis if (inclusion criteria): panelists were patient representatives, relatives or part of the relevant occupational group (multi-professional experts in the field, reported at least ≥5 years of experience in dealing with desire to die and/ or suicidality, and reached high scores in self-assessed confidence and knowledge with desire to die and/ or suicidality (rating at least four on a 0 to 6 Likert scale on one of the four items). Data from the Delphi survey was analyzed statistically for quantitative results using SPSS 23 (IBM Corp., Armonk, NY, USA). Thematic analysis was applied to analyze free text comments. An a priori criterion of 80% agreement among panelists was defined to determine consensus [30] on the importance of a section, requiring a rating of at least four on a one to five Likert scale.

Further details on sampling, data collection and analysis are reported in the study protocol [28].

## Results

### Patient interviews

Between 9/2017 and 1/2018, 11 (69%) patients receiving palliative care participated in face-to-face interviews lasting nine to 79 minutes. Reasons for patients refusing to partake or health professionals judging the patient incapable of participation were (acute) deterioration of physical (3/5) or mental (2/5) health status. Table 1 presents further details on interviewees.

**Table 1:** Sociodemographic data of the interviewees

*N*	11
Age	
*M*, *SD*	71.7 (13.9)
*Min, Max*	51, 89
Gender	
Male (*n*, %)	7 (63.6)
Female (*n*, %)	4 (36.4)
First language	
German (*n*, %)	11 (100.0)
Educational level	
Higher education entrance qualification (n, %)	5 (45.5)
Higher secondary school (*n*, %)	5 (45.5)
Lower secondary school (*n*, %)	1 (9.1)
Vocational training	
Professional training (*n*, %)	6 (54.5)
University degree (*n*, %)	2 (18.2)
None (*n*, %)	2 (18.2)
Not specified (*n*, %)	1 (9.1)
Diagnosis	
Cancer (colon, lung, liver, breast & abdominal, lower jaw, larynx, glioblastoma) (*n*, %)	7 (63.6)
Geriatric multimorbidity (*n*, %)	2 (18.2)
Chronic obstructive pulmonary disease (COPD) (*n*, %)	2 (18.2)
Care Setting	
Home care (*n*, %)	4 (36.4)
Residential care (*n*, %)	2 (18.2)
Hospice care (*n*, %)	3 (27.3)
In-patient care (*n*, %)	2 (18.2)
Interview Setting	
At home (*n*, %)	4 (36.4)
Hospice (*n*, %)	3 (27.3)
Residential care facility (*n*, %)	2 (18.2)
Hospital (*n*, %)	2 (18.2)

All but one of the interviewees reported desire to die either as a wish for hastened death or as acceptance of death without requiring to hasten it. A desire for physician assisted suicide was reported explicitly once, and two interviewees preferred death to deteriorating symptoms. Isolation, the feeling of being a burden, hopelessness and fear of pain were reasons reported for desire to die.

Deductive categories based on topics from the interview guideline were complemented with inductive coding of the interviews, producing five main categories with various sub-categories: “Actively building the relationship”, “Conversation partner”, “Conversation context and structure”, “Conversation set up and framework” and “Own Desire to Die”. Within the formulated categories, patients mainly confirmed the dimensions and recommendations of the clinical approach draft, yet emphasized individuality:

*Well, you can’t develop a very RIGID guideline, I think. It’ll need to cover a vast spectrum, starting from one point at the bottom and spreading VERY, VERY wide apart at the top.* (patient 1)

However, some sub-categories added valuable insight and contributed to the revision of the semi-structured clinical approach draft. Those sub-categories are reported below.

Almost all of the interviewees appreciated the **proactive assessment of desire to die** by health professionals:

Interviewer: *Would it have been helpful to you, if [the health professional] had addressed [the desire to die]?*

Patient: *Yes. I think so. [ … ] I don’t know how other patients feel, but talking about it was very difficult for me.* (patient 3)

To initiate and discuss problems, establishing a **trustful health-professional-patient-relationship** is a prerequisite, which both sides have to allow for. Unobtrusively signaling an appreciative attitude best frames the setting: conscious eye contact and relational touch can help to establish intimacy, if appropriate:

*Who among you [health professionals] even gives hugs anymore or takes someone’s arm? [ … ] You all have a hard time with that.*

Interviewer: *This would be something important to you?*

Patient: *Of COURSE.* (patient 7)

When arranging for an **appropriate environment** to talk about desire to die, privacy and the patient’s mental state should be taken into account. Furthermore, taking enough time was unanimously appreciated and considered helpful in signaling special attention.

One patient reported that thoughts concerning hastened death primarily arose upon first confrontation with the cancer diagnosis. Breaking bad news was also experienced as traumatic by other cancer patients.

While most of the patients preferred to talk to their physician, all members of the palliative care team were deemed fit for a dialogue about desire to die. If required, participants indicated that **support from external experts** should be sought:

*[The health professional said:] “We can call a pastoral worker for you [ … ] to talk to.” [ … ] I didn’t KNOW what to talk about with a pastoral worker. But he was here for an hour and there WAS a lot to talk about, apparently.* (patient 5)

### Delphi survey

Based on the clinical approach draft revised after the patient interviews, a Delphi survey was conducted between 1/2018 and 3/2018. Round one was open for 22 days; round two for 16 days. There was almost no dropout (5.0%) between rounds; during the first round 210 invitees participated and 200 during round two. We excluded 61/51 panelists that did not meet inclusion criteria from analysis. Therefore, in both rounds, 149 participants were fit for data analysis according to inclusion criteria. This number goes far beyond our original recruitment plans, encompassing 50–70 panelists and is due to the overwhelming feedback from German (nursing) experts. With 91% of the sample reporting to work in direct patient contact, it consists largely of practitioners. For socio-demographic details on the Delphi sample see Table 2.

**Table 2:** Sociodemographic data of the Delphi sample

*N*			149
Age	*Mean* (*Minimum, Maximum)*		49.3 (19, 72)
			***n (%)***
Gender	Female		107 (71.8)
	Male		42 (28.2)
Residence	Germany		132 (88.6)
	Other countries		17 (11.4)
	Spain		➢ *n* = 5
	Canada		➢ *n* = 3
	Switzerland, Norway		➢ *n* = 2 each
	USA, Australia, El Salvador, Sweden, Portugal		➢ *n* = 1 each
Expertise^a^	Nursing		91 (61.1)
	Physician		21 (14.1)
	Psychology and psychotherapy		9 (6.0)
	Spiritual care		11 (7.4)
	Ethics counseling		10 (6.7)
	Social work		1 (0.7)
	Relatives		12 (8.1)
	Research and science		20 (13.4)
	Non-practitioners, e.g. moral philosophers		13 (8.7)
	Other		17 (11.4)
**Self-assessment**			
			*n* (%)
Experience in years	Dealing with desire to die (DD) in clinical practice	< 1	3 (2.0)
		1–9	58 (38.9)
		≥ 10	81 (54.4)
		missing	7 (4.7)
	Dealing with suicidality in clinical practice	< 1	39 (26.2)
		1–9	41 (27.5)
		≥ 10	63 (42.3)
		missing	6 (4.0)
	Studying DD from a theoretical perspective	< 1	58 (38.9)
		1–9	61 (40.9)
		≥ 10	21 (14.1)
		missing	9 (6.0)
	Studying suicidality from a theoretical perspective	< 1	82 (55.0)
		1–9	39 (26.2)
		≥ 10	19 (12.8)
		missing	9 (6.0)
			*Mean* (*Standard Deviation)*
Confidence^b^	Dealing with DD		4.16 (1.00)
	Dealing with suicidality		2.92 (1.37)
Knowledge^b^	DD		3.98 (1.07)
	Suicidality		2.97 (1.36)

^a^Multiple responses possible

^b‘^0’ (‘not confident at all’) to ‘6’ (‘very confident’) Likert scale adapted from Morita (2007) [25]

#### Suitability and usefulness

87% of the respondents (*n* = 129) valued the structure of the clinical approach as ‘(very) suitable’, 95% (*n* = 141) rated the clinical approach to be’(very) useful’ for everyday clinical practice.

#### Importance of individual domains

Table 3 displays the results of the importance ratings for Delphi round one and two.

**Table 3:** Clinical approach domains and Delphi survey importance ratings

	*Mean* (*Standard Deviation*)			Consensus^a^		
	Round 1^b^	Round 2^c^	*p*	Round 1	Round 2	Increase
**A – Usage Notes**						
1. Usage notes^d^	–	4.32 (0.91) [5]	–	–	**92.6%**	–
**B – Conversation Aspects**						
2. Actively building the relationship	4.64 (0.85)	–	–	**92.6%**	–	–
3. Proactively addressing desire to die	4.01 (0.94)	4.16 (0.92)	**< 0.05**	74.5%	**83.2%**	8.7%
4. Closure of discussion	4.62 (0.74)	–	–	**92.6%**	–	–
5. After discussion	4.64 (0.65)	–	–	**94.0%**	–	–
**C – Classification, Meaning and Functions**						
6. Classification of desire to die	4.26 (1.0)	4.37 (0.80)	0.10	85.2%	**90.6%**	5.4%
7. Background and meanings of desire to die	4.81 (0.50)	–	–	**97.3%**	–	–
8. Functions of desire to die	4.31 (1.07)	4.64 (0.73)	**< 0.01**	83.9%	**95.3%**	11.4%
**D – (Self-)Reflection**						
9. Conscious engagement with own attitudes and emotions	4.77 (0.53)	–	–	**97.3%**	–	–
10. Self-protection	4.74 (0.53)	–	–	**96.0%**	–	–
**E – Further Recommended Action**						
11. Further recommended action	4.53 (0.85)	4.68 (0.56)	0.07	87.9%	**95.3%**	7.4%

^a^ Likert scale items were labeled ‘5’ (‘very important’) to ‘1’ (‘not important at all) with the option to report ‘don’t know’ (exclusion from analysis). Consensus was assumed if participants rated domains with ‘4’ (‘quite important’) or ‘5’ (‘very important’). Percentages are quotas of all participants who answered a respective question, not of the entire sample

^b^ For all ratings the full range of possible answers was used except for ‘conscious engagement with own attitudes and emotions’ (*Min* = 2, *Max* = 5) and ‘self-protection’ (*Min* = 3, *Max* = 5)

^c^ For all ratings the full range of possible answers was used expect for ‘further recommended action’ (*Min* = 2, *Max* = 5)

^d^ Domain added after round 1

For all domains except ‘proactively addressing desire to die’, the a priori consensus criterion (≥80% agreement) was met during the first Delphi round (see Table 3). As only four domains did not reach 90% of agreement, we took reaching < 90% to indicate potential for optimization and therefore asked our panelists to reevaluate all such domains (proactively addressing, classification, functions, further recommended action) in the second round. During round two, all domains except ‘proactively addressing desire to die reached an agreement ≥90%. As our panelists opposed too narrow prompts on how to address desire to die, we changed all interrogative clauses and prompts in the original draft into the instructions and circumscriptions found in the final version of the semi-structured clinical approach (see Additional file 2). Other modifications based on Delphi results are reported in Table 4.

**Table 4:** Modifications of the semi-structured clinical approach based on Delphi comments

Contents of comments	Implementation^**a**^
free text answers across all domains pointed to the need to provide general notes on proper usage of the clinical approach	added a new domain: ➪ ‘usage notes’
suggestion on asking whether patients think about terminating life prematurely criticized as being too direct	added a new suggestion: ➪ ‘Explore thoughts related to not wanting to live anymore’
clinical approach seen to be at danger of provoking checklist type of interrogation due to bullet point setup	changed interrogative clauses to instructions: ➪ ***‘Exists or existed***Explore fear of death and dying?’ ➪ ***‘Exists or existed***Explore thoughts related to terminating life prematurely***?***’
complexity and changeability of desire to die in palliative patients seen to run counter to unambiguous classification	added a new suggestion: ➪ ‘In general, keep in mind: desire to die is complex and prone to change’
“manipulate” in the respective function of desire to die seen to be poor choice of words	changed wording: ➪ ‘Attempting to ***manipulate***influence family or health professionals’
“attracting attention” in the respective function of desire to die seen to be poor choice of words	changed wording: ➪ ‘***Attracting***Drawing attention to oneself and one’s trouble’
“treatment contracts” seen as bad practice, especially when involving handshakes for sealing the contract as it seemed to suggest “clean hands practice”	changed wording, rated old and new version during round 2: ➪ ‘Entering into a treatment contract with handshake in cases of latent suicidality’ (32.9% agreement, *M* = 3.00, *SD* = 1.19) ➪ ‘Entering into a treatment ***contract with handshake***agreement in cases of latent suicidality in order to win time for interventions’ (79.2% agreement, *M* = 4.17, *SD* = 0.96)
suggestion on passive euthanasia seen as poorly worded	changed wording: ➪ Letting die (passive euthanasia) as a legal option (foregoing, restriction or cancellation of life sustaining and life prolonging measures)’
selection of therapeutic approaches listed as examples in respective suggestions seen as too narrow	added a new suggestion during round 2: ➪ ‘Offering other (psycho-)therapeutic interventions (e.g. family therapy, psychotherapy, art therapy)’ summed up all related suggestions into one for the finalized clinical approach: ➪ ‘Offering counseling or (psycho-)therapy for individuals or groups’

^a^plain text: same wording in round 1 and 2; bold italic: deletions; underlined: additions

No significant statistical differences in ratings for all domains were found among subsamples (e.g. gender, expertise, self-assessments).

#### Controversial aspects

Aside from the relatively moderate agreement on ‘proactively addressing desire to die’ during the Delphi process, classifying desire to die as thoughts of terminating life prematurely, wish for assisted suicide or euthanasia in the clinical approach was also met with concern. In the final version of the semi-structured approach, these classifications were nonetheless included but go hand in hand with introductory information that points out the broadness of the desire to die phenomenon. The introductory information in Additional file 3 shows this in more detail.

## Discussion

### (Proactively) addressing desire to die within trustful health professional-patient-relationships

Since desire to die may be a potential way to cope with advanced disease at some point during the disease trajectory [33], exploring it and allowing its emotional expression in conversation may be beneficial to all patients. A desire to die can be expressed in different ways and proactively addressing it may help to clarify reasons more openly and at an earlier stage. However, in clinical practice, desire to die is not yet routinely assessed by health professionals and talking about it is associated with discomfort [23].

Concerns about adverse effects of discussing desire to die, e.g. triggering suicidal thoughts in patients, are widespread [23] and became apparent in our Delphi survey. Current studies confirm that asking about suicidality causes no harm but may reduce experienced burden and distress [34]. Based on preliminary studies [23, 35] these findings can likely be extrapolated to palliative care. Currently, our clinical approach is only available for health professionals attending the related multi-professional training program. Within this training, we present the aforementioned findings and the broad definition of desire to die prior to handing out the semi-structured clinical approach. This is meant to alleviate concerns about iatrogenic risks of addressing desire to die.

A recent descriptive study employed an ad hoc semi-structured clinical interview for proactive assessment of a wish to hasten death among advanced cancer patients [35]. Participants did not experience this as distressing, but considered it important regardless of whether they were personally affected. The patients we interviewed indicated that they appreciated the initiation of desire to die conversations by health professionals. (Proactively) addressing the issue in this way can open communication about patients’ emotions, even in absence of desire to die [13], which may help to build a trusting relationship that can help to preserve the will to live [36] and perhaps diminish suicidality. The German National Ethics Advisory Board recommends suicide prevention programs to counteract requests for assisted suicide and the importance of communication is stressed for suicide prevention [37]. These have been the reasons for including the recommendation that desire to die should be (proactively) addressed in the 2019 edition of the German palliative care guideline for patients with incurable cancer [6]. Establishing and maintaining a relationship are essential in addressing patients’ desire to die [23], so that the establishment of a therapeutic relationship should precede conversations about desire to die. Therefore, the semi-structured clinical approach places a special focus on actively building relationships.

### Framing, timing and patient-attunement of communication

Although patients do not object to enquiries about potential desire to die, even when they do not personally have one [35], the impact is likely to depend on *how and when* such conversations take place. Various guidelines have been developed for the improvement of health professional-patient-communication in palliative care settings, e.g. “NURSE – Naming, Understanding, Respecting, Supporting, Exploring” for difficult conversation tasks in oncology [38] and “SPIKES – A Six-Step Protocol for Delivering Bad News” for breaking bad news [21]. An evaluation of the SPIKES implementation found that privacy positively influence patients’ rating of their knowledge gained and the experience of the amount of time devoted to it as being sufficient [39]. This is consistent with our interview results that the framing of conversations is of particular importance. As receiving bad news was experienced as traumatic and a trigger for desire to die by some of our interviewees suffering from cancer, it may be appropriate to delay this discussion to follow-up conversations.

As has been found regarding breaking bad news in oncology [40] and in light of the feedback from our Delphi panelists, the clinical approach is designed to promote a patient-centered procedure, giving suggestions on how to address potential desire to die while avoiding checklist-type interrogations [41]. As it is also stated in the approach’s usage notes, there is no need to address desire to die in the same way for all patients and not all thematic aspects need to be addressed in every conversation about desire to die.

### Appropriate (therapeutic) responses towards desire to die

The first intervention advised with respect to every patient with desire to die is the initiation of an open communication about it. The semi-structured clinical approach suggests before considering any other action, health professionals should aim to understand the patient’s wish, its backgrounds, meanings and functions. As severe physical symptoms are also related to desire to die [42], optimal symptom control is especially important in patients expressing such desire. Discussion of the expected disease trajectory and options for treatment can also decrease anxiety [43]. For patients who experience physical suffering as unbearable, sensitively pointing out that therapy withdrawal or palliative sedation until death are legal possibilities to alleviate suffering. Its administration needs to be tailored towards the individual’s needs and in sensitive coordination with relatives [6]. Pointing out these and other options early on can be part of a more general discussion of a patients’ Advance Care Plan (ACP). ACP, the discussion about possible further treatment or discontinuation of treatment for severe illnesses, is provided in Germany according to §132 g social security code V. Up to now, the national statutory health insurance system has only financed the ACP for those insured who are undergoing inpatient care in nursing homes [44]. It has been implemented increasingly [45] in palliative care regardless of setting with up to 44% of people aged 60 and older reporting the completion of an ACP [46]. As of today, there is no standard procedure for the implementation of ACP. It can vary across situations and should be seen as a dynamic, continuous process including all involved parties. A recent qualitative review of the international literature points out that patients unanimously prefer ACP discussions being proactively initiated by health professionals [47]. These characteristics of ACP are consistent with our approach to proactively addressing desire to die.

Diagnostic clarification of depression is indispensable as it has been shown to predict [42] and moderate [48] desire to die. Suicidality with completed suicide at its most extreme can be a manifestation of desire to die, yet is not necessarily to be equated with it. Therapeutic interventions such as Meaning-Centered Therapy [49] and a supportive expressive therapy named Managing Cancer and Living Meaningfully (CALM) [50] have been shown to alleviate depressive symptoms in patients with advanced cancer [49, 51, 52].

### Controversy concerning physician-assisted suicide and euthanasia

Our Delphi panelists expressed only moderate agreement for directly addressing wishes for euthanasia or assisted suicide. Since euthanasia (§216 national criminal code) and physician-assisted suicide (§217 national criminal code) were forbidden in Germany at the time of data collection, participants may have preferred to avoid naming such wishes because their realization would be illegal. Another concern expressed by our Delphi panelists was that acknowledging desire to die could pressure patients into actively seeking hastened death, maybe by seeking help in neighboring countries with a less restrictive legal situation [18]. Both scenarios point to a fundamental conviction that the question of the desire to die causes the emergence of such desires. This view can lead health professionals to avoid talking about it. We emphasize the importance of a clear distinction between *acknowledging* and *agreeing with* or even endorsing and supporting desire to die.

Also, the legal situation in Germany and its controversial discussion is duly addressed in the mandatory training course that introduces the semi-structured clinical approach as a practical tool. The repeal of §217 (the prohibition of physician-assisted suicide by the Federal Court of Justice in February 2020^20^ does not end the controversial discussion about it. A statement issued by the German Society for Palliative Medicine opposes the repeal [53]. However, it is also emphasized that allowing open and respectful conversations about desire to die will always remain vital regardless of changes in the legal context [53]. We fully agree with this last statement: allowing room for patients to talk about their desire to die and supporting health professionals in dealing with it is important independent of legal and therefore also of national context. The developed semi-structured clinical approach offers that support.

### Self-protection and self-care

In addition to recommendations aimed at improving the patients’ well-being, protecting the well-being of health professionals is also important. Conversations about desire to die can be enriching but might also cause emotional stress [54]. It is therefore important for health professionals to develop a sensitive grasp of their own feelings and to protect themselves from emotional overload [54]. In order to manage discussions of desire to die appropriately, health professionals should also be aware of own values, norms and their personal stance concerning death wishes. Supervision, case meetings and everyday peer exchange can help to deal with stress or difficult situations [55].

### Study limitations

With 16 invited and 11 interviewed patients, the sample may appear small. However, a sample size of 10–20 is both common and suitable for a qualitative analysis [56, 57], if textual saturation is achieved. Due to usual difficulties in the recruitment of palliative patients [58] and limited resources, a convenience sampling strategy was applied, yielding a low heterogeneity in diagnoses of our interview partners. As 64% of them were cancer patients the results appear applicable to oncological settings, but the generalizability of findings onto other palliative patients may be limited. Although we aimed to compose a balanced sample of research and clinical practice perspectives for our Delphi survey, we ultimately recruited mostly practitioners, only a small number of relatives and no patient representatives. Participating health professionals were multi-professional experts with more than 60% of them being nurses. Statistical analyses displayed no significant differences between professions. However the numbers in each profession may not have been sufficient to detect such differences.

### Clinical implications

As health professionals are frequently confronted with desire to die, a semi-standardized communication guide for dealing with desire to die has great potential for clinical practice. The fact that more practitioners participated in the Delphi survey than originally planned shows their great interest in the topic and its particular relevance in palliative care. The semi-structured clinical approach thus became a tool tailored directly towards everyday practice of people working in the field. This can include all professions directly in contact with palliative patients provided they have partaken in the mandatory training course: physicians, nurses, psychologists, social and spiritual care workers as well as volunteers. We expect the semi-structured clinical approach in conjunction with our training program to foster multi-professional competencies across all health care structures, especially on dealing with desire to die in patients with serious health-related suffering due to severe illness.

## Conclusions

The major achievement of this study is the creation of the first consensus-based semi-standardized approach for (proactively) assessing and optimally responding towards desire to die based on literature review, patient interviews and expert consent. Besides recommendations for communication, the semi-structured clinical approach lists possible types, meanings and functions of desire to die as well as feasible interventions. Also health professionals’ self-reflection on own attitudes and emotions concerning desire to die as well as self-protection are taken into consideration.

## Supplementary information

**Additional file 1.** Semi-structured interview guideline; Interview guideline with all questions for patient interviews.

**Additional file 2.** Semi-structured clinical approach for addressing Desire to Die; Finalized version of the clinical approach after revision through patient interviews and consensus finding through Delphi process.

**Additional file 3.** Introductory information to the semi-structured clinical approach; complementary information on the clinical approach not consented in the Delphi process.

## Data Availability

The datasets generated during and/or analyzed during the current study are not publicly available, but are available from the corresponding author on reasonable request.

## Abbreviations

ACP: Advance Care Planning
CALM: Managing cancer and living meaningfully
COREQ: Consolidated criteria for reporting qualitative research
CREDES: Guidelines for Conducting and reporting Delphi Studies in palliative care
DDRS: Desire for Death Rating Scale
SAHD: Schedules of Attitudes for Hastened Death
StGB: National criminal code
