# Supplementary material for: The desire to die in palliative care: a sequential mixed methods study to develop a semi-structured clinical approach
Source: BMC Palliat Care. 2020 Apr 16;19:49. doi: 10.1186/s12904-020-00548-7 (PMC7164236; doi:10.1186/s12904-020-00548-7)
Supplement: Supplementary file 1 — Additional file 1. Semi-structured interview guideline; Interview guideline with all questions for patient interviews. [file 12904_2020_548_MOESM1_ESM.docx]

**DEDIPOM semi-structured interview guideline (Phase 1)**

**Introduction**

Mutual conversation between patients and health professionals constitute an essential part of therapy and companionship. Especially in conversations with patients with severe, incurable disease, personal subjects arise and oftentimes the approaching of the end of one’s life is thematic. I want to address a special topic in this context.

| **Getting started** | **Some patients with a severe disease wish that their life might end sooner rather than later. If you put yourself in a situation such as this, what do you think are the prerequisites that make it possible to talk about subjects as difficult as desires to die? What in important during such a conversation?** |
| --- | --- |
| Proactively Adressing DD | - - How would you feel if a member of the treatment team would initiate a conversation about a potential desire to die?     - E.g. being asked by a physician whether you have a desire to die   - How important is it to you that you could speak with someone of the treatment team about your potential desires to die? |
| **Composition** | **How do you think should the conversation be structured ideally?** |
| Content  Structure | - - What contents should the conversation focus on? What should be talked about?     - E.g. reasons, therapeutic options, thoughts related to desire to die   - How should the conversation be structured?   - Should the health professional address the topic directly or would it be better if they placed it within the context of other topics? |
| **Conversation aspects** | **(Provided you had a desire to die) With whom could you imagine having a conversation about it?** |
| Personality  Relationship | - - Is there anything that would make it easier for you to entrust a health professional with your desire to die?     - E.g. a trustful relationship?     - What signals would help you / what signals should the health professional be sending?   - Are there any special skills or personal qualities that such a person should have? |
| **Own Desire to Die** | **Did you ever wish for death to come faster or life to end sooner since your diagnosis?** |
|  | - - Did you talk so someone about those wished? With whom?     - - If not: Why not?       - If yes: Questions at the end of the interview guideline |
| **Miscellaneous** | **What else might be helpful for such a conversation from your perspective?** |
|  |  |
| **End of the interview** | **Is there something else you might want to add? E.g. something that is important to you and has not been addressed yet?*** |
|  | - - Is there something that you want to know or that remains unclear?   - How did you experience the interview?   - What made you take part in this interview?   - Information about possibility for phone contact on the next day (or later) |
| **Thanks** | **We sincerely thank you for your participation. The interview results will surely help in the improvement of conversations between patients and health professionals.** |

*If the patient is under a lot of strain…

… there ist he possibility to ask about what is difficult, but also about what is good. The latter should definitely get its necessary space. This procedure ensures that crises are neither evaded, nor that the conversation does not end with a phase experienced as a crisis. .^[[1]](#footnote-1)^

***Exemplary formulation:***

1. „What did you experience as consoling since your diagnosis?“

🡪Take up ressources from preceding conversation

| **Preceding**  **conversations** | **Can you tell me how this conversation went?** |
| --- | --- |
| - - What did you talk about?   - How did you experience the conversation?     - What impact did the conversation have on you?   - How did you experience your conversation partner?   - What was good about the conversation? What could have been better / what could the HP have done better?   - Did the HP initiate the conversation or did a statement you made start the conversation? (Did you utter a DD? How? How was the reaction?)   - Was there more than one conversation? | |

1. Rosenthal, Gabriele ; Loch, Ulrike: Das Narrative Interview. In: Schaeffer, Doris (Ed.) ; Müller-Mundt, Gabriele(Ed.): Qualitative Gesundheits- und Pflegeforschung. Bern u.a. : Huber, 2002. - ISBN 3-456-83890-5, pp. 221-232. URN: <http://nbn-resolving.de/urn:nbn:de:0168-ssoar-57670> [↑](#footnote-ref-1)
