# Supplementary material for: The desire to die in palliative care: a sequential mixed methods study to develop a semi-structured clinical approach
Source: BMC Palliat Care. 2020 Apr 16;19:49. doi: 10.1186/s12904-020-00548-7 (PMC7164236; doi:10.1186/s12904-020-00548-7)
Supplement: Supplementary file 2 — Additional file 2. Semi-structured clinical approach for addressing Desire to Die; Finalized version of the clinical approach after revision through patient interviews and consensus finding through Delphi process. [file 12904_2020_548_MOESM2_ESM.docx]

**Clinical approach for addressing desire to die**

**A – Usage Notes**

| - Conversations on desire to die are always unique and shaped by the persons, their relationships and surrounding situations; they do not have a predetermined order or structure. - Information provided in the clinical approach is not meant to be worked through like a checklist but is meant to promote an open and non-judgmental dealing with desire to die. - Some of the information provided in the guide such as recommendations concerning the classification or functions of desire to die may serve as important background information for health professionals but need not necessarily be discussed with patients explicitly. |
| --- |

**B – Conversation Aspects**

| **Actively building the relationship** | - Meeting the patient with concern and respect - Listening actively and without interrupting the patient - Accepting the patient and their suffering - Encouraging the patient to express emotions - Using simple language that is understandable for the patient - Reacting caringly, compassionately and sensitively in language and gestures - Enduring silence - Signaling availability to communicate even when difficult topics or conflicts arise - Maintaining the relationship even through challenging situations - Establishing physical contact, e.g. holding a hand (if appropriate) - Indicating that time is available (and how much) - Providing a setting as comfortable as possible (location, privacy) |
| --- | --- |
| **Proactively addressing desire to die** | - Exploring fears of death and dying - Exploring whether the patient experiences their distress as unbearable - Exploring thoughts related to not wanting to live anymore - Exploring thoughts related to ending life prematurely |
| **Closure of discussion** | - Inquiring about personal and social resources - Summarizing the most important points - Inquiring about uncertainties and further need to talk - Realistic assurance of willingness and availability to talk - Arranging an additional date for conversation if desired - Pointing out that team members will be informed confidentially |
| **After discussion** | - Filing in patient documentation - Informing other team members - Reflecting on the course of the conversation, relationship and emotions |

**C – Classification, Meaning and Functions**

| **Classification of desire to die** | - In general, keep in mind: desire to die is complex and prone to change. - Patient’s acceptance of death without the wish to hasten death - Current wish to hasten death - Possible (hypothetical) wish to hasten death - Desire to die in the form of assisted suicide (e.g. by being provided with medication) - Desire to die in the form of active euthanasia (e.g. by being given medication) - Thoughts about and intent of taking one’s life (suicidality) |
| --- | --- |
| **Background and meaning of desire to die** | - Desire to die as reaction to… - … physical suffering - … social problems such as isolation or conflicts - … loss of dignity as perceived by the patient - Desire to die as expression of… - … depressed mood - … fear - … hopelessness - … suffering related to meaning in life (spiritual and existential suffering) - … feeling to be a burden - … a complicated course of disease |
| **Functions of desire to die** | - Acute call for help - Means of talking about death and dying - Attempt to regain autonomy and control - Attempt to influence family or health professionals - Drawing attention to oneself and one’s suffering |

**D – (Self-)Reflection**

| **Conscious engagement with own attitudes and emotions** | - Being mindful of own emotions - Being mindful of own behavior in language and gestures - Reflecting own emotions with the team - Accepting that intensity of subjective suffering differs from person to person - Being mindful of one’s own attitudes towards desire to die - Being mindful of one’s expression of one’s own attitudes - Being mindful that one’s attitude influences the patient’s reaction and the course of the conversation - Realistically assessing one’s personal and professional qualification |
| --- | --- |
| **Self-protection** | - Being mindful of what one is able and willing to provide - Getting informed about the legal situation - Accepting contradictory remarks (ambivalence) about desire to die as a possible part of processing - Being mindful of signs of own distress - Being mindful of own fears - Being mindful of own triggers - Using opportunities for own relief - Seeking support from colleagues and superiors - Seeking internal or external supervision |

**E – Further Recommended Action**

| - Recognizing depression and treating it with state of the art practices (e.g. S3 guidelines) - Seeking medical-psychiatric assistance in cases of acute suicidality - Entering into a treatment agreement in cases of latent suicidality in order to win time for interventions - Offering “palliative sedation” (10 points EAPC) in case of unbearable pain - Letting die (“passive euthanasia”) as a legal option (foregoing, restriction or cancellation of life sustaining and life prolonging measures) - Offering counseling or (psycho-)therapy for individuals or groups - Seeking case-based ethics counselling, when divergent opinions occur within the team |
| --- |
