# Supplementary material for: The desire to die in palliative care: a sequential mixed methods study to develop a semi-structured clinical approach
Source: BMC Palliat Care. 2020 Apr 16;19:49. doi: 10.1186/s12904-020-00548-7 (PMC7164236; doi:10.1186/s12904-020-00548-7)
Supplement: Supplementary file 3 — Additional file 3. Introductory information to the semi-structured clinical approach; complementary information on the clinical approach not consented in the Delphi process. [file 12904_2020_548_MOESM3_ESM.docx]

Introductory Information on the Clinical Approach

Patients that receive palliative care frequently express desires to die in one way or another. Health professionals are often insecure about how to handle that. This semi-structured clinical approach was developed within the scope of a training program on dealing with desire to die at the University Hospital Cologne (Germany). The clinical approach is meant to provide security and orientation when dealing with openly or covertly expressed desires to die.

The clinical approach is an essential part of the training program and can be used to recognize and (proactively) address desires to die. When doing so, patients and their desires to die should be treated with outmost respect. In the ensuing conversation, the health professional and the patient together try to uncover the meanings of such wishes.

The development of the clinical approach was funded (among others) by the *Bundesministerium für Bildung und Forschung (BMBF)*. Over the course of several projects that built up on each other, the clinical approach has been developed and modified into its current shape. To this end, we asked patients, their relatives and national as well as international experts for their perspectives.

This booklet contains the clinical approach as developed based on our research. All recommendations therein are not to be taken as the last word on the matter or to encompass all that there is to it. They are proposals that should be supplemented and advanced in their practical application.

The prerequisite for the sensible and adequate use of the clinical approach is the participation in a related training program. The clinical approach does not contain a list of recommended readings, as the relevant literature is presented during the training.
